# Greyscale and Paper Electrochromic Polymer Displays by UV Patterning

**DOI:** 10.3390/polym11020267

**Published:** 2019-02-05

**Authors:** Robert Brooke, Jesper Edberg, Xavier Crispin, Magnus Berggren, Isak Engquist, Magnus P. Jonsson

**Affiliations:** 1 Laboratory of Organic Electronics, Department of Science and Technology, Linkoping University, SE-601 74 Norrkoping, Sweden; robert.brooke@ri.se (R.B.); jesper.edberg@ri.se (J.E.); Xavier.crispin@liu.se (X.C.); magnus.berggren@liu.se (M.B.); Isak.engquist@liu.se (I.E.); 2 RISE Acreo, ICT Department, Printed Electronics, Research Institutes of Sweden, Acreo, 601 17 Norrkoping, Sweden

**Keywords:** conductive polymers, PEDOT, patterning, electrochromic, electrochromic displays, paper displays, digital cellulose, cellulose, paper electronics, electrochromism, vapor phase polymerization

## Abstract

Electrochromic devices have important implications as smart windows for energy efficient buildings, internet of things devices, and in low-cost advertising applications. While inorganics have so far dominated the market, organic conductive polymers possess certain advantages such as high throughput and low temperature processing, faster switching, and superior optical memory. Here, we present organic electrochromic devices that can switch between two high-resolution images, based on UV-patterning and vapor phase polymerization of poly(3,4-ethylenedioxythiophene) films. We demonstrate that this technique can provide switchable greyscale images through the spatial control of a UV-light dose. The color space was able to be further altered via optimization of the oxidant concentration. Finally, we utilized a UV-patterning technique to produce functional paper with electrochromic patterns deposited on porous paper, allowing for environmentally friendly electrochromic displays.

## 1. Introduction

Electrochromic devices have important applications in various fields, including in smart windows for efficient temperature regulation in buildings [1,2,3], smart packaging, and information technology [4,5]. Recently, commercial products have emerged containing electrochromic displays, such as smart windows, or to provide information to the user, such as battery life or other visual/graphic representations of information [6,7].

However, electrochromic displays have so far been limited in their application, partly due to the difficulties involved in patterning the materials at the micrometer scale using high throughput methods [8]. The techniques developed for patterning electrochromic conductive polymers (CPs) are highly promising in that respect, particularly in combination with these CPs’ excellent stability, good optical contrast and exceptional optical memory for low power consumption [8,9,10]. These attributes show high potential for commercial CP-based electrochromic products. Furthermore, high-quality films of these otherwise insoluble materials can be prepared by vapor phase polymerization (VPP). While other vapor deposition systems exist, such as chemical vapor deposition [11] and molecular layer deposition [12], VPP has been shown to provide CP films with excellent properties [13,14,15].

Poly(3,4-ethylenedioxythiophene) (PEDOT) has been the most studied CP for electrochromic applications [16,17,18,19]. In addition to its deep blue reduced state and transparent oxidized state, PEDOT shows good mechanical and electrochemical stability and provides sufficiently high conductivity (>1000 S/cm) to facilitate its own switching without need for supporting current collectors [14,16]. PEDOT’s electrochromic behavior is suitable for many applications, ranging from smart windows to smart package displays [20]. Several reports have recognized that the possibility to pattern PEDOT could significantly extend its functionality and application potential [17,21]. While dip-pen lithography and other techniques have achieved submicrometer patterning resolution, they generally require expensive equipment and long processing times [22]. More established patterning techniques for large area production, like screen printing, instead possess other drawbacks such as relatively thick films (>1 µm) and limited resolution (>100 µm) [4,17].

We have developed a promising alternative technique that combines UV-light mediated patterning and VPP [23,24]. In brief, this new patterning technique locally alters the chemical environment of an oxidant film by UV-irradiation through a photolithography mask. Patterned CP films are then synthesized via VPP by exposing the irradiated films to monomer vapor, followed by washing and drying. The method enables high resolution patterning of PEDOT (<10 µm) over areas that are only limited by the size of the photolithography mask. Moreover, the technique can be used to modulate the optical properties of the produced polymer to form PEDOT images that can disappear and reappear by switching the redox states of the CP. This feature is related to the high optical contrast between UV-treated and pristine PEDOT in the reduced state, while the optical contrast between UV-treated and pristine areas is low in the oxidized state. Previous work has demonstrated the possibility of creating picture-to-picture electrochromic displays using multiple types of CPs [24]. However, the devices were “black and white” only (binary patterns with sharp edges and no gradients) and showed very slow switching (>5 min), which was probably related to having two different types of CPs layered on the same ITO-coated substrate and using another ITO-coated substrate as a counter electrode, which provided an insufficient counter charge. 

Here, we present a new concept that enables electrochromic “greyscale” (patterns with gradients) picture-to-picture devices with rapid-switching, based only on PEDOT with tosylate (Tos) as the counter ion. We demonstrate that locally varying the UV-dose by exposure through a greyscale mask produces greyscale PEDOT:Tos pictures, which appear and disappear upon switching. Separating two films with imprinted PEDOT:Tos pictures with a gel-electrolyte allowed picture-to-picture switching, with only one of the images shown at a time. VPP parameter variation enabled control of the quality and transparency of the imprinted pictures. 

Flexible organic devices hold great promise for various disposable electronic applications such as medical tests and smart labels. However, today such devices are typically produced on plastic substrates, which could contribute to the microplastic pollution of the planet. To enable more environmentally friendly electronics, we adapted the UV-patterning technology to be used on cellulose-based substrates, creating electrochromic paper devices. The study thereby also shows progress in the field of paper electronics, highlighting the potential of the UV-patterning technique for sustainable and recyclable products based on cellulose materials that are lightweight and easily processable, and which provide excellent flexibility and robustness.

## 2. Materials and Methods

Clevios^TM^ CB 40 (40 wt % Fe(III) p-toluenesulfonate (Fe(Tos)_3_) in butanol) was purchased from Heraeus (Hanau, Germany). The 3,4-ethylenedioxythiophene (EDOT), 1-ethyl-3-methylimidazolium ethyl sulfate (EMIM-ES), hydroxyethyl cellulose (HEC) and the triblock co-polymer poly(ethylene glycol)-block-poly(propylene glycol)-block-poly(ethylene glycol) (PEG-PPG-PEG, 5800 g·mol^−1^) were purchased from Sigma Aldrich (St. Louis, MO, USA). All chemicals were used as received without further purification. Solutions were prepared in ambient conditions without the need for a glove box. The VPP was performed in a vacuum hotplate chamber (JP Selectra S.A, Barcelona, Spain). 

The procedure for the VPP of EDOT was similar to that previously reported [15], where the oxidant solution was composed of Fe(Tos)_3_ (12.3 wt % unless otherwise stated) and PEG-PPG-PEG (23.1 wt %) in a 1.2:3 mixture of ethanol and butanol. The oxidant solution was created by mixing PEG-PPG-PEG with Fe(Tos)_3_ in butanol and ethanol. The solution was stirred and heated to approximately 50 °C in order to melt and dissolve the PEG-PPG-PEG. The oxidant solution was used immediately after formation, although it is also useable for months after formation with no decrease in the polymer properties.

The oxidant solution was spin-coated onto cleaned (thoroughly with isopropanol) glass substrates (with gold electrodes for electrical characterization), ITO-coated glass or PET, at 1500 rpm for 30 s. The oxidant-coated substrates were placed on a 70 °C hot plate for 30 s before undergoing UV-light exposure for 900 s using a UV-lamp with an intensity of 20 mW/cm^2^ at 405 nm and 10 mW/cm^2^ at 365 nm. For the patterning, photomasks printed on PET plastic substrates were created by printing black greyscale images on transparent overhead projector substrates using a standard office printer. The photomasks were mounted on quartz glass and suspended above the oxidant-coated substrates prior to UV-light exposure to create regions with varying exposure. EDOT VPP was performed at −70 mmHg of pressure at a temperature of 60 °C for 30 min. After polymerization, the thin films were washed with ethanol and air-dried. 

Electrochromic behavior for individual films was investigated by encapsulating a PEDOT:Tos pattern on transparent ITO-glass with a counter electrode of bare ITO-glass and with a gel-electrolyte between the electrodes. This enabled investigation of the individual electrochromic behavior without influence from the counter electrode. The ITO counter electrode could provide sufficient charge for visibility testing. Picture-to-picture electrochromicci devices were constructed using patterned PEDOT:Tos films as both the working and the counter electrochromic electrodes. The gel electrolyte was made by mixing 7 wt % HEC in EMIM-ES ionic liquid and stirring using a magnet bar while heating the mixture to 100 °C on a hotplate. 

**Figure 1:**
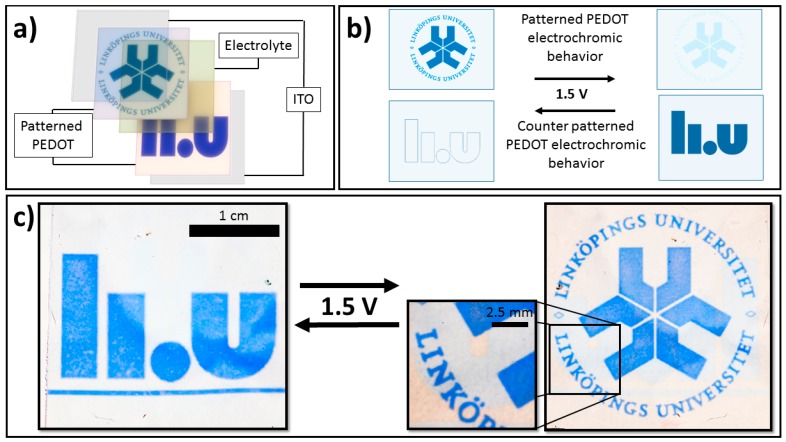
(**a**) Picture-to-picture electrochromic device architecture based on two patterned poly(3,4-ethylenedioxythiophene with tosylate (PEDOT:Tos) layers separated by a transparent electrolyte. (**b**) Schematic illustration of the individual electrochromic behavior of the two patterned PEDOT layers when voltages of 1.5 V were applied in the forward and reverse bias. The images appeared and disappeared in opposite voltage directions, allowing for a picture-to-picture device. (**c**) Photographs of the same picture-to-picture electrochromic device in both states, showing clearly the new (left) and old (right) Linköping University logos. The device can be seen switching in real time in SIMovie1. The thickness of the PEDOT:Tos layers were approximately 200 nm for the pristine regions and approximately 350 nm for the ultraviolet (UV)-exposed regions.

Prototype electrochromic devices were created by doctor blade coating the gel-electrolyte onto the CP ITO electrode and placing the second CP pattern ITO electrode on top, as depicted in Figure 1a. The devices were switched by applying a voltage of 1.5 V at varying polarity. 

Proof-of-concept paper displays were created using a lift-off technique with the conductive polymer patterns in water. The PEDOT:Tos patterns were then transferred to porous paper with another global layer of PEDOT:Tos deposited on the backside of the paper to act as the counter electrode. The paper was soaked with the gel-electrolyte and sandwiched between two pieces of ITO-coated glass.

Color contrast measurements were performed on PEDOT:Tos samples using a spectrophotometer (Datacolor Mercury, Lucerne, Switzerland). A 5 cm × 5 cm PEDOT:PSS on PET was employed as the counter electrode in a lateral electrochromic setup to avoid interference from the counter electrode. The larger area of the counter electrode ensures that enough charge can be supplied to the working electrode for complete electrochemical switching to occur. The electrochemical switching was performed in 1M NaCl aqueous solution while applying an electrical bias of ±1.5 V between the working and counter electrodes. The samples were switched between their oxidized and reduced states five times before performing the measurements. The samples were then switched to their oxidized/reduced state and dried with a nitrogen gun before performing the color measurements in reflection with the samples positioned on a white back-scattering background. L*, a* and b* (color space coordinates) values were recorded for the oxidized and reduced states of pristine and UV-light treated PEDOT films. Color contrast between the two states or samples (denoted 1 and 2) were calculated from the color space coordinates using the formula [25]:

$$\sqrt{{\left(L_{1}^{*}-L_{2}^{*}\right)}^{2}+{\left(a_{1}^{*}-a_{2}^{*}\right)}^{2}+{\left(b_{1}^{*}-b_{2}^{*}\right)}^{2}}.$$

Film thickness was measured using atomic force microscopy (AFM). AFM images were obtained in tapping mode using a Veeco Dimension 3100 (Bruker, MA, USA). The thickness measurements were analyzed using the Gwyddion software (Brno, Czech Republic).

## 3. Results and Discussion

Figure 1a depicts the general device architecture for the presented UV-patterned picture-to-picture displays. Two individually patterned PEDOT layers were separated by a transparent electrolyte, such that they will be in the opposite redox states regardless of the direction of electrochemical switching. 

Figure 1b illustrates this feature schematically, with only one of the two images (the one on the currently reduced PEDOT:Tos film) being visible for each switching direction. As further discussed below, this concept makes use of the fact that the optical contrast in the image is high for the reduced film but low for the oxidized film, which forms the basis of the picture-to-picture display. Figure 1c demonstrates the concept for a real device, with switching between the old (right) and new (left) logotypes of Linköping University when switching the polarity of the voltage (1.5 V) applied over the electrochromic device. Since the PEDOT:Tos films were positioned on opposite sides of the electrochromic device, one was reduced when the other was oxidized and vice versa. This allowed only one picture (the reduced side) to be visible when the voltage was applied in a certain direction. Reversing the polarity made the other picture appear while the first picture disappeared. Furthermore, the two PEDOT:Tos films acted as each other’s counter electrodes, which enabled rapid-switching. The device was able to switch between the two logos within seconds (<10 s, see SIMovie1), which is acceptable for most electrochromic applications and is a great improvement when compared with previous designs (≈300 s) [24]. The modification of electrochromic behavior has been discussed in our previous work and is thought to be related to a reduced ability of UV-exposed regions to accommodate charges. Further detail on how UV-exposing the oxidant affects the fundamental and electrochromic properties of CP films (e.g., conductivity, absorbance, etc.) can be found in our previous work [23,24].

The patterned pictures above were produced using black and white (binary) photomasks and we could further extend the concept to demonstrate greyscale (gradient) picture-to-picture displays by using greyscale photomasks. These photomasks not only contain regions that allow all or no UV-light to pass, but also regions that partially block UV-light to different degrees. Since the degree of color modulation in the polymer film is proportional to the amount of UV-irradiation, exposing the oxidant film through this type of mask makes it possible to imprint greyscale images onto the final PEDOT:Tos films. 

**Figure 2:**
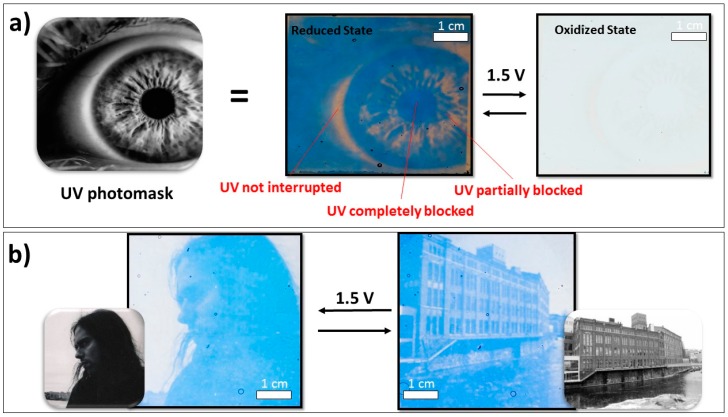
(**a**) Greyscale picture used as the UV photomask to allow gradients to form within the PEDOT pattern. The result allows pictures to be patterned into PEDOT:Tos films and are shown to appear and disappear in the reduced (middle panel) and oxidized (right) states, respectively. (**b**) Electrochromic device switching between a portrait of a former lab member and our office building at Linköping University (greyscale UV mask insets). The device was fabricated with two PEDOT:Tos patterned pictures using the electrochromic device architecture illustrated in Figure 1a.

Figure 2a shows examples of photographs of such greyscale PEDOT:Tos pictures of an eye (middle) together with the corresponding photomask (left). As marked in the figure, the final UV-patterned PEDOT:Tos image contains regions of varying optical properties depending on the degree of local UV exposure. To investigate the electrochromic properties of the greyscale images, the PEDOT:Tos film was incorporated into an electrochromic device with bare ITO-glass as the counter electrode. As seen in Figure 2a, the picture was clearly visible in the reduced state of the film (middle), while it disappeared when the film was switched to its oxidized state (right). This demonstrates that greyscale UV-patterned pictures can also be turned on and off by changing the oxidation state of the polymer, which allowed for the construction of greyscale picture-to-picture displays as well as for binary pictures (based on the same configuration as shown in Figure 1a). Figure 2b shows photographs of such a device for both switching polarities, together with the corresponding photomask images. Each picture appeared when the corresponding PEDOT:Tos layer was in its reduced state, while the other (oxidized) picture is not visible. Further examples of greyscale picture-to-picture devices can be seen in Figure S1. 

After demonstrating the general concept of greyscale picture-to-picture switching, the means of tuning the details of the pictures were investigated. Varying the oxidant concentration was shown to be a practical way of dramatically affecting the PEDOT:Tos film thickness and therefore also the image transparency and contrast. 

**Figure 3:**
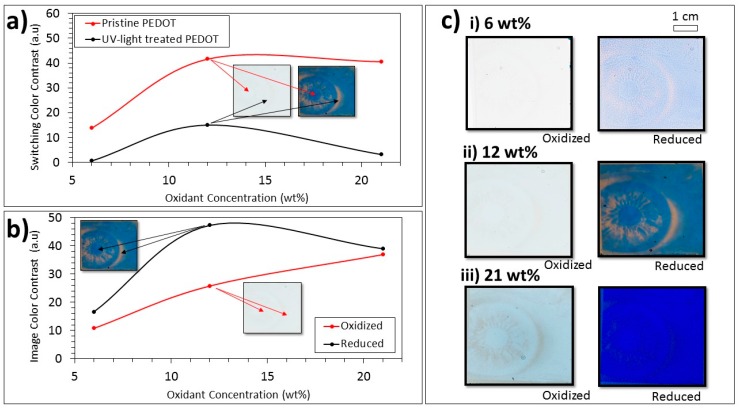
(**a**) Switching color contrast values (oxidized vs reduced PEDOT) at different oxidant concentrations comparing pristine and UV-light-treated PEDOT. The arrows and insets illustrate the types of regions that are compared in these graphs (representation only). (**b**) Image color contrast values (pristine PEDOT versus UV-light-treated PEDOT) at various oxidant concentrations when the films were oxidized and reduced. The arrows and insets illustrate what is compared in the two graphs (representation only). (**c**) Photographic comparison between the oxidant concentration samples encapsulated using bare ITO for individual electrochromism accompanied by color contrast representations of the switching behavior of the images.

Figure 3a shows the measured switching of color contrast values (see details in the Methods section) between the oxidized and reduced states of the PEDOT:Tos films produced using different oxidant concentrations. Results are presented for both UV-treated (black) and pristine (red) films for three different oxidant concentrations. We note that the UV-treated films showed drastically reduced switching contrast compared with pristine films. This finding corroborates previous reports [24]. The values in Figure 3b instead correspond to the color contrast between the pristine and UV-light treated films, for both the oxidized (red) and reduced (black) states. Hence, this parameter provides an estimation of the image contrast in the respective redox states. While the image color contrast for the oxidized state increased gradually with oxidant concentration, the image color contrast for the reduced state, as well as the switching color contrast for both the UV-irradiated and pristine films, initially increased with the oxidant concentration but decreased at yet higher concentrations. These findings are related to the increase in film thickness with oxidant concentration [26] and to the absorptivity of the different materials. For very low oxidant concentrations, all films and states were thin and relatively transparent, resulting in low contrasts. All contrast values therefore initially increased with increasing oxidant concentrations and film thickness. For the other extreme, with very thick films, all films would be opaque, which would also result in low contrast. As explained in our previous work [18], the highest contrast is therefore obtained at an optimum thickness that depends on the absorption coefficients of the materials. This explains why the image contrast for the comparatively absorptive reduced film (black in Figure 3b) decreased between 12 and 21 wt %, while the contrast for the more transparent oxidized film (red in Figure 3b) was still increasing in this range. Photographs of UV-patterned greyscale images also illustrate this reasoning. The image contrast for the reduced state clearly increased with oxidant concentration in the whole measured range (left column in Figure 3c). Applications requiring images to completely disappear (in the oxidized state) may therefore benefit from patterns made from low oxidant concentrations. By contrast (pun unintended), the photographs in the right column of Figure 3c clearly show that the contrast for the reduced state was highest for 12 wt % compared with both 6 and 21 wt %. In addition, the difference in contrast between reduced and oxidized states was highest for 12 wt %, and the oxidized image was hardly visible. Hence, 12 wt % forms suitable conditions for high contrast images that can repeatedly appear and disappear upon switching. This concentration was used for all images in the other figures of this paper. L*a*b* values (color space coordinates) for all samples and redox states can be found in Table S1. 

**Figure 4:**
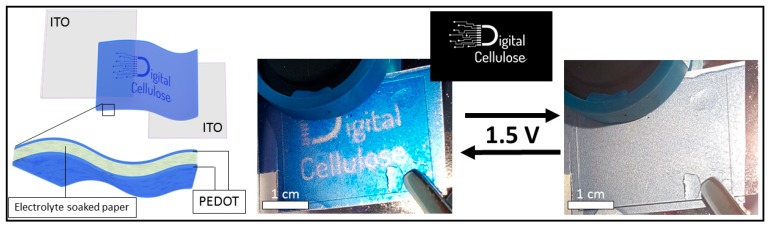
Schematics and photographs of paper electrochromic devices using electrolyte-soaked filter paper.

Finally, we demonstrate that the UV-light patterning technique enabled the production of greyscale electrochromic pictures on cellulose (paper)-based substrates. Such paper devices are difficult to fabricate with many other patterning techniques, due to soaking/wicking, stretching and general disconnection in the porous fiber-based material. Direct polymerization onto paper resulted in the penetration of the oxidant solution into the fiber structure, which resulted in thick polymer films with poor optical transparency and contrast. To avoid polymerization inside the paper substrates, we used a novel lift-off technique. PEDOT:Tos films were first patterned on glass substrates following the standard procedure. When put in water, the films delaminated from the substrate and could be transferred to highly porous filter paper. This technique allowed films with well-defined sub-micrometer thickness to be added to cellulose films while preserving the porosity of the fiber/fibril structure. The filter paper coated with UV-patterned PEDOT:Tos was used as the mechanical support and as the separator for a paper display, as seen in Figure 4. A PEDOT:Tos counter electrode was placed on the other side of the paper and soaked with electrolyte to allow electrochromic functionality. PEDOT:Tos films can switch electrochromically without the use of an additional conducting layer [16], however, the current films were rather fragile and easily scratched when directly contacted with conventional external electrodes. We therefore used ITO-coated glass as a contact for these proof of concept devices. Future devices may use flexible substrates with a conducting stripe to serve as a robust contact between the PEDOT films and an external electrode. 

## 4. Conclusions

In summary, this report focused on progressing the UV-light mediated VPP patterning of CPs for novel electrochromic device applications. We demonstrated a new concept for electrochromic picture-to-picture devices with rapid-switching based on two patterned PEDOT films. The use of greyscale photomasks for the patterning step enabled an extension of the concept to gradient pattern electrochromic picture-to-picture displays. We investigated switching and image color contrasts for different starting oxidant concentrations, finding optimum conditions at intermediate concentrations for pictures that can appear and disappear upon electrochromic switching. Finally, we reported a flexible paper-based electrochromic display based on the UV-patterning technique, thus highlighting the technique’s potential to contribute to sustainable optoelectronic applications. 

## Supplementary Materials

The following are available online at http://www.mdpi.com/2073-4360/11/2/267/s1, Figure S1: Electrochromic device architecture and further examples of complex patterns within electrochromic devices. Table S1: Complete L*a*b* color space values of the as prepared, oxidized and reduced pristine PEDOT and UV-light treated PEDOT with the oxidant concentration varied. SIMovie1: Switching of the two Linkoping University logotypes.
