# Supplementary material for: Greyscale and Paper Electrochromic Polymer Displays by UV Patterning
Source: Polymers (Basel). 2019 Feb 5;11(2):267. doi: 10.3390/polym11020267 (PMC6419265; doi:10.3390/polym11020267)
Supplement: Supplementary file 1 [file polymers-11-00267-s001.zip › polymers-417495-Supplementary Materials/Brooke-Polymers 2019-Greyscale and Paper Electrochromic Polymer Displays by UV Patterning-Supplemental-SI.pdf]

Supporting Information for

Greyscale and Paper Electrochromic Polymer Displays by UV Patterning

Robert Brooke[1,2], Jesper Edberg[1,2], Xavier Crispin[1], Magnus Berggren[1], Isak Engquist[1], Magnus P. Jonsson[1*]

[1] Linkoping University, Department of Science and Technology, Laboratory of Organic Electronics, SE-601 74 Norrkoping, Sweden

[2] RISE Acreo, ICT Department, Printed Electronics, Research Institutes of Sweden, Acreo, 601 17, Norrkoping Sweden

* Correspondence: magnus.jonsson@liu.se

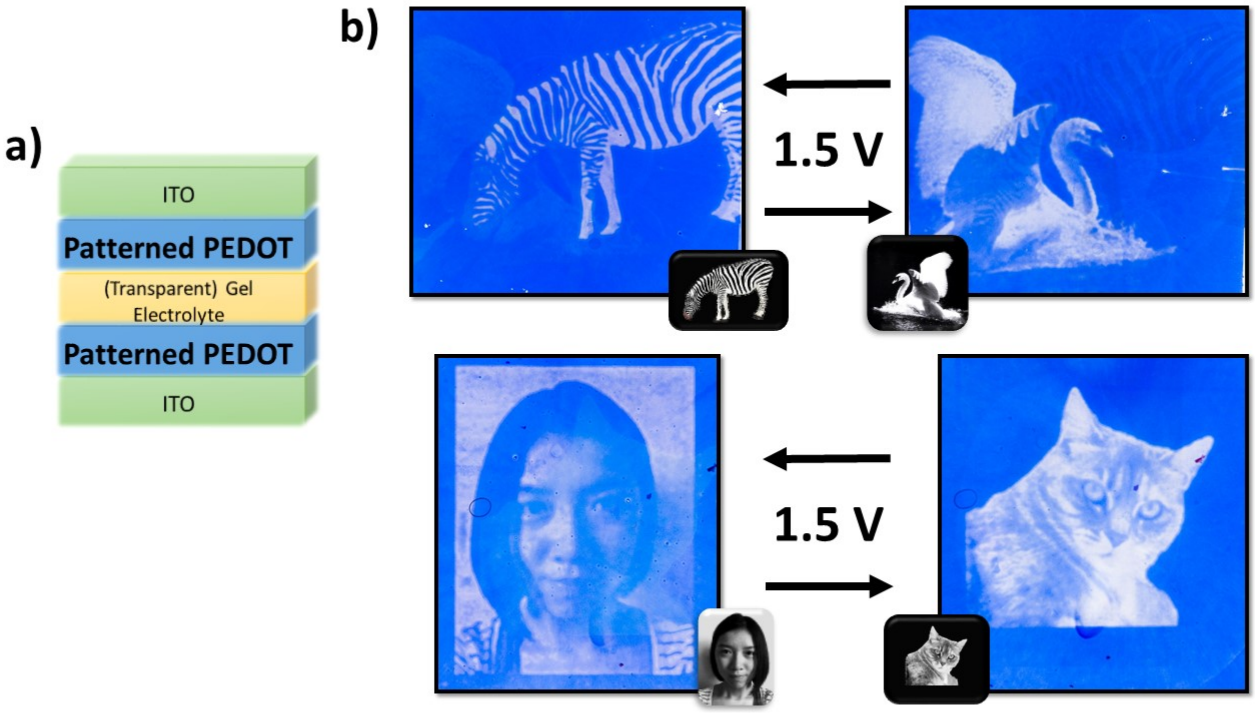

Figure S1. Electrochromic device architecture and further examples of complex patterns within electrochromic devices.

Table SI1. Complete L*a*b* color space values of the as prepared, oxidized and reduced pristine PEDOT and UV-light treated PEDOT with the oxidant concentration varied.

Sample	Redox state	L*	a*	b*
**Pristine PEDOT**				
6 wt%	As prepared	71.15	-3.72	-11.62
6 wt%	Oxidized	71.86	-3.94	-9.34
6 wt%	Reduced	59.94	0.46	-14.91
12 wt%	As prepared	49.41	-6.07	-28.09
12 wt%	Oxidized	52.9	-4.94	-27.24
12 wt%	Reduced	28.9	16.68	-53.53
21 wt%	As prepared	23.7	-4.97	-36.72
21 wt%	Oxidized	27.0	-1.69	-38.02
21 wt%	Reduced	3.09	31.12	-38.02
**UV-light treated PEDOT**				
6 wt%	As prepared	68.72	-0.04	-0.86
6 wt%	Oxidized	68.49	0.26	0.09
6 wt%	Reduced	68.45	0.38	-0.63
12 wt%	As prepared	44.16	-1.43	-6.32
12 wt%	Oxidized	46.93	-3.22	-2.17
12 wt%	Reduced	35.52	4.49	-8.25
21 wt%	As prepared	15.29	-4.06	-12a.74
21 wt%	Oxidized	10.3	5.32	-5.77
21 wt%	Reduced	8.45	7.15	-7.66
